# Virtual insulin pump initiation is safe effective in children adolescents with type 1 diabetes

**DOI:** 10.3389/fcdhc.2024.1362627

**Published:** 2024-04-30

**Authors:** Benjamin Udoka Nwosu, Margaret Pellizzari, Maia N. Pavlovic, Jason Ciron, Rashida Talib, Rubab Sohail

**Affiliations:** ^1^ Division of Endocrinology, Department of Pediatrics, Cohen Children’s Medical Center, Hempstead, NY, United States; ^2^ Department of Pediatrics, Donald and Barbara Zucker School of Medicine at Hofstra/Northwell, Queens, NY, United States; ^3^ Biostatistics Unit, Office of Academic Affairs, Northwell Health, NY, United States

**Keywords:** type 1 diabetes, hemoglobin A1c, COVID-19 pandemic, children, adolescents, insulin pump, continuous glucose monitoring

## Abstract

**Objective:**

There is no head-to-head comparison of the safety and efficacy of virtual versus in-office insulin pump initiation for youth with type 1 diabetes in the US. The study’s aim was to determine the safety and efficacy of virtual versus in-office pump initiation in pediatric type 1 diabetes.

**Research design and methods:**

A longitudinal retrospective study of 112 subjects: 65% (n=73), ages 11.2 ± 3.8 years(y), received in-office training; and 35% (n=39), ages 12.0 ± 4.0y, received virtual training. The number of White subjects was 40 (55%) in the in-office group, and 25 (66%) in the remote group; while Black subjects were 11 (15%) in the in-office group and 4 (10%) in the virtual group. Data were collected at pump initiation, 3 and 6 months.

**Results:**

There were no significant differences in sex, race, height, weight, BMI, and the duration of diabetes between the groups at baseline. There was no significant difference in A1c between the groups at 0, 3, and 6 months. A1c correlated significantly with the glucose management indicator at 0, 3, and 6 months: baseline: r=0.49, p<0.0001; 3 months: r=0.77, p<0.0001; and 6 months: r=0.71, p<0.0001. There was no relationship between A1c or TIR and pubertal status, BMI, sex, or race. A1c was significantly elevated in the non-White individuals at 6 months only: 57.9 mmol/mol (50.8-69.4) versus 51.9 mmol/mol (46.5-59.6)], p=0.007.

**Conclusion:**

Virtual insulin pump initiation is safe and effective in children with type 1 diabetes. This approach could accelerate the adoption of the use of diabetes technology in minority populations in the US.

## Introduction

Type 1 diabetes is a disease marked by persistent hyperglycemia secondary to autoimmune destruction of the pancreatic β-cells (1). The use of diabetes technology such as continuous subcutaneous insulin infusion, also known as the insulin pump, continuous glucose monitors, and their integration into automated insulin delivery (AID) systems are associated with improved glycemic management in children and adolescents with type 1 diabetes (2). The AID systems consist of combinations of glucose control algorithms, glucose sensors, and insulin pumps that work seamlessly to maintain glycemia, increase time in range (TIR), and reduce A1c, hypoglycemia, and hyperglycemia (3).

Traditionally, the process of training and initiation of insulin pumps occurs in the clinic or hospital setting. However, the COVID-19 pandemic and the associated lockdowns and social distancing made it very difficult to conduct these insulin pump initiation training sessions in the clinic or medical offices (4).

In a bid to continue the delivery of exceptional diabetes care to patients at our Diabetes Center following the implementation of the COVID-19 lockdown in New York on March 23, 2020, we initiated a virtual pump initiation program for families that wanted to transition to insulin pumps from multiple daily injections, while maintaining social distancing. With the return to the post-pandemic period, it becomes necessary to determine the safety and efficacy of the measures employed during the pandemic to determine their suitability for ongoing diabetes care, as well as tools for future pandemic preparedness.

This is crucial as there is a dearth of data on the efficacy and safety of virtual insulin pump initiation in children with type 1 diabetes. Results from studies in adult subjects with type 1 diabetes who were upgraded to AID before and during the COVID-19 pandemic suggested that the glycemic outcomes of virtual pump training were comparable to those of in-person training (5). However, no pediatric study has reported a head-to-head comparison of the safety and efficacy outcomes of virtual versus in-person training for children and adolescents with type 1 diabetes.

The primary aim of this study was to determine the safety and efficacy of virtual insulin pump teaching and initiation compared to the standard in-office teaching and insulin pump initiation. The secondary aim was to determine the factors, such as race, weight, age, and sex that could explain any differences between the two insulin pump initiation strategies.

## Research design and methods

### Ethics statement

The protocol for this retrospective study was approved by the Institutional Review Board of Northwell Health. Subjects’ medical records were anonymized and de-identified prior to analysis.

### Subjects

The patient population was comprised of 118 pediatric patients with a confirmed diagnosis of type 1 diabetes from the Cohen Children’s Medical Center Database of Northwell Health, New York, USA. Of these, 6 patients who underwent a combined in-office and virtual training module were excluded, and only 112 subjects were included in the analysis. The pediatric endocrinology clinic treats approximately 1000 patients with type 1 diabetes. The inclusion criteria were a diagnosis of type 1 diabetes by American Diabetes Association criteria (6) as detailed below, male and female sex, age of 3-16 years, ongoing use of CGM, and the initiation of an insulin pump from 01/02/2020 to 12/29/2021. The diagnosis of type 1 diabetes was based on any of the following criteria: fasting blood glucose of ≥ 126 mg/dL (7 mmol/L), and/or 2-hour postprandial glucose of ≥200 mg/dL (11.1 mmol/L), and/or random blood glucose of ≥200 mg/dL with symptoms of polyuria and/or polydipsia. All subjects had one or more diabetes-associated auto-antibodies, namely zinc transporter-8 autoantibodies, islet cell cytoplasmic autoantibodies, glutamic acid decarboxylase antibodies, insulinoma-associated-2 autoantibodies, and insulin autoantibodies. Subjects were excluded if they had other forms of diabetes mellitus. Anthropometric and demographic data were collected at the time of insulin pump initiation. Glycemic data were collected at 0, 3, and 6 months.

### In-office and virtual training modules

The virtual training module was directed by diabetologists who were supported by certified diabetes education specialists and nutritionists. Virtual sessions were performed using the Zoom video conferencing application (Zoom Video Communications, San Jose, California).

All subjects used CGM before their transition from multiple daily injections to continuous subcutaneous insulin infusion (CSII), also known as insulin pumps. Participants who used the T:slimx2 with Control IQ had an algorithm while those on Omnipod EROS or DASH used no algorithm. The insulin pump transition module consisted of 3 training sessions on a general introduction to insulin pumps and how to safely use the devices with their existing continuous glucose monitors (7). The first session was the Pump Basics Class, which was a 60-minute session that focused on reviewing clinical scenarios that could arise while using the insulin pump. These clinical scenarios include hypoglycemia, hyperglycemia, ketosis, instances of pump failure, and when to call the diabetes center. The second session was the Saline Start Class during which the pump reservoir was filled with sterile normal saline for use in practice mode for one week. This allowed the patients and their families to familiarize themselves with the workings of the pump. The third session was the Insulin Start Class. During this class, the pump device was loaded with insulin for the first time and the patient discontinued subcutaneous insulin injections. Each class involved a practical demonstration of the mechanics of the pump and its features, standard didactic instructions, and the use of a teach-back technique to ensure understanding and retention of instructions (8). Follow-up care involved weekly phone communication with the families by certified diabetes care and education specialists and nutritionists. The diabetes team monitored real-time CGM-derived glucose levels on cloud repositories and made follow-up phone calls to ensure competency. If needed, additional training sessions were conducted to ensure that the families were confident in using the device.

The in-person module also consisted of 3 training sessions that were conducted according to standard protocol. Here the pump instructions were provided to the patient in the clinic. The patient and their family then returned to the clinic for the initiation of the saline phase. Upon successful completion of the saline phase, the patient was then transitioned to the insulin phase, and glucose data were uploaded to the cloud repository.

### Anthropometry

The approach to anthropometry has been previously described in detail (9). Weight was measured to the nearest 0.1 kg using an upright scale. Height was measured to the nearest 0.1 cm using a wall-mounted stadiometer that was calibrated daily. BMI was calculated from the formula: weight/height^2^ (kg/m^2^), and expressed as z-score for age and sex, based on National Center for Health Statistics (NCHS) data (10). Overweight was defined as BMI of ≥85^th^ but <95^th^ percentile, and obesity was defined as BMI of ≥95^th^ percentile for age and gender.

### Glycemic data capture

For both groups, baseline A1c data were obtained either at the time of initiation of the pump or 1-2 months before pump initiation. Subsequent A1c data were obtained at 3 months and 6 months either as a point-of-care A1c or venous A1c drawn at local Northwell laboratories for close correlation with the Northwell point-of-care A1c values.

Continuous glucose metric data were downloaded from cloud repositories and data for the 2 weeks preceding each 3-month visit were used to generate the CGM metric for the particular visit. The CGM used was Dexcom G6 and the web-cloud platforms were Dexcom Clarity (https://clarity.dexcom.com/professional/), Glooko (https://my.glooko.com/users/sign_in) and t:connect (https://tconnecthcp.tandemdiabetes.com/hcp_account/#/hcplogin. Medical charts were reviewed for either diabetes complications or records of hospital admissions for the management of diabetes complications such as diabetic ketoacidosis and severe hypoglycemia.

### Assays

The assay techniques for A1c and diabetes-associated autoantibodies have been previously described (9).

### Statistical analyses

Descriptive statistics were reported by insulin pump training initiation group (in-person, virtual) for demographics and clinical variables at baseline (age at diagnosis (years), gender (female, male), race (White, Black or African American, Other), insulin pump type (Omnipod, t:slimx2 with Control IQ), height (cm), height z-score, weight (kg), weight z-score, BMI (kg/m2), BMI z-score, BMI percentile, A1c, time in range (TIR), and glucose management indicator (GMI), a predictor of A1c level based on 14-day continuous glucose monitor (CGM) data. Continuous variables were reported as means and standard deviations (SD) if normally distributed, and as medians and interquartile ranges (IQ: 25th percentile, 75th percentile) if non-normally distributed. Categorical variables were reported as frequencies and percentages. A 2-sample t-test was used to test for differences in continuous characteristics between the 2 training groups for normally distributed data and a Wilcoxon signed rank test for non-normally distributed data. A chi-square or Fisher’s exact test was used to test the significance of categorical characteristics between the 2 groups, as appropriate. Spearman correlation coefficients were calculated between GMI and A1c levels at each time point. All analyses were performed using available data. For all analyses, results yielding p-values <0.01 through a Bonferroni-like adjustment were considered statistically significant. This lower p-value threshold was chosen to reduce the probability of type 1 error from the multiple tests being performed. All analyses were conducted using SAS version 9.4 (SAS Institute Inc., Cary, NC).

## Results

### Anthropometry and demographics

There were no differences in sex, race, height, weight, BMI, and the duration of type 1 diabetes between the groups at baseline (**Table 1**). There was a predominance of White subjects in both groups: 55% (n=40) for the in-office group, and 66% (n=25) for the remote group. Black individuals were 15% (n=11) in the in-office group, and 10% (n=4) in the remote group. The rest of the participants were classified as Other. While 100% (n=39) of the remote group used the Omnipod pump, 44% (n=32) of the in-office group used the Omnipod pump while 56% (n=41) used the t:slimx2 with Control IQ insulin pump.

**Table 1:** Baseline characteristics of the subjects (n=112).

Parameter	In-office/Hospital Initiation N = 73	Virtual/Remote Initiation N = 39	p-value*
Age at pump initiation, years [mean (SD)]	11.2 (3.8)	12.0 (4.0)	0.33
Sex Male	49 (67.1)	19 (48.7)	0.06
Race White individuals Black individuals Other	40 (54.8) 11 (15.1) 22 (30.1)	25 (65.8) 4 (10.5) 9 (23.7)	0.63
Pump Type Omnipod T:slimx2 with Control IQ	32 (43.8) 41 (56.2)	39 (100) 0 (0)	<0.0001
Height, cm	151.8 (133.5 – 165.5)	153.10 (137.30 – 165.0)	0.95
Height z-score [(mean (SD)]	-1.10 (1.26)	-1.33 (1.37)	0.36
Weight, kg	43.80 (29.30 – 61.80)	45.0 (33.0 – 60.80)	0.68
Weight z-score [(mean (SD)]	-0.55 (1.96)	-0.69 (1.67)	0.51
BMI, kg/m^2 ƪ^	18.73 (17.12 – 22.43)	20.03 (17.19 – 22.40)	0.54
BMI z-score ^ƪ^	0.16 (-0.88 – 1.09)	0.27 (-0.34 – 0.81)	0.73
BMI percentile	74.0 (46.0 – 89.0)	79.50 (59.0 – 92.0)	0.24
Baseline A1c (%)	7.50 (6.70 – 8.20)	7.30 (6.40 – 8.30)	0.98
Baseline A1c (mmol/mol)	58.5 (49.7-66.1)	56.3 (46.5-67.2)	0.98
Baseline GMI ^ƪ^	7.35 (6.85 – 8.10)	7.25 (6.60 – 7.90)	0.30
Baseline TIR (70 – 180 mg/dl) ^ƪ^	62.95 (44.80 – 77.0)	62.60 (44.0 – 82.0)	0.78
Duration between diabetes diagnosis and pump training initiation (years)	0.70 (0.40 – 1.80)	0.70 (0.30 – 2.40)	0.53

BMI, Body Mass Index; GMI, Glucose Management Index (%); TIR, Time in Range (%).

*p-values calculated using Wilcoxon rank-sum test, Kruskal-Wallis, two-sample t-test, Chi-square test or Fisher’s exact test, as appropriate. For sex, race, and pump type, summary statistics are reported as frequencies and percentages. For continuous variables, summary statistics are reported as medians (1^st^ quartile – 3^rd^ quartile), unless otherwise stated. ^ƪ^ Sample size for the following variables: i. BMI = 110; ii. BMI z-score = 108; iii. BMI percentile = 111; iv. Baseline GMI = 110; v. Baseline TIR = 110.

The comparison of fast-acting insulin use in the subjects is as follows: Humalog (40 in-person, 21 virtual), Novolog (15 in-person, 6 virtual), Admelog (14 in-person, 10 virtual), Lispro (3 in-person, 2 virtual), and Aspart (1 in-person, 0 virtual). There were no significant differences in the fast-acting insulin types between the groups (p=0.87).

### Glycemic control

**Figure 1:**
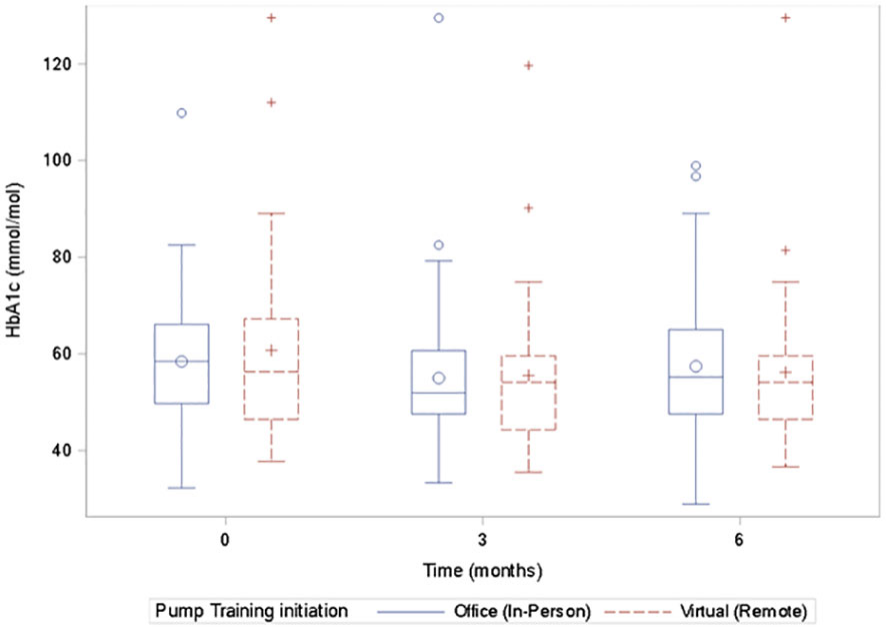
Box plots of the A1c values for the virtual and the in-person groups at 0, 3, and 6 months showing no significant difference in the A1c values at each time point between the groups.

There was no significant difference in A1c between the virtual and in-person groups at 0, 3, and 6 months (**Figure 1**). A1c and the GMI correlated significantly at all time points, 0, 3, and 6 months as follows: baseline: r=0.49, n=110, p<0.0001; 3 months: r=0.77, n=98, p<0.0001; and at 6 months: r=0.71, n=105, p<0.0001.

When the subjects were stratified by age into <10 years and >10 years, there was no significant difference between the groups for A1c and TIR. Similar stratification by BMI status into normal-weight (BMI of <85^th^ percentile) or overweight or obese (BMI of ≥85^th^ percentile) did not show any difference between the groups for both A1c and TIR. There was equally no significant difference in glucose variability and time above range (TAR) between the 2 groups at any point.

A within-group analysis comparing baseline and 6-month glucose data showed no significant differences between baseline and 6 months in glucose variability, TIR, A1c, and GMI in either group.

A comparison of changes in A1c and TIR between the 39 subjects who started on the Omnipod insulin pump virtually and the 32 subjects who were started on the Omnipod insulin pump in person showed no significant difference in A1c at baseline, p=0.4; and at 6 months, p=0.09. There were equally no differences in TIR, p=0.6 at baseline, and p=0.3 at 6 months.

### Glycemic control and race

**Figure 2:**
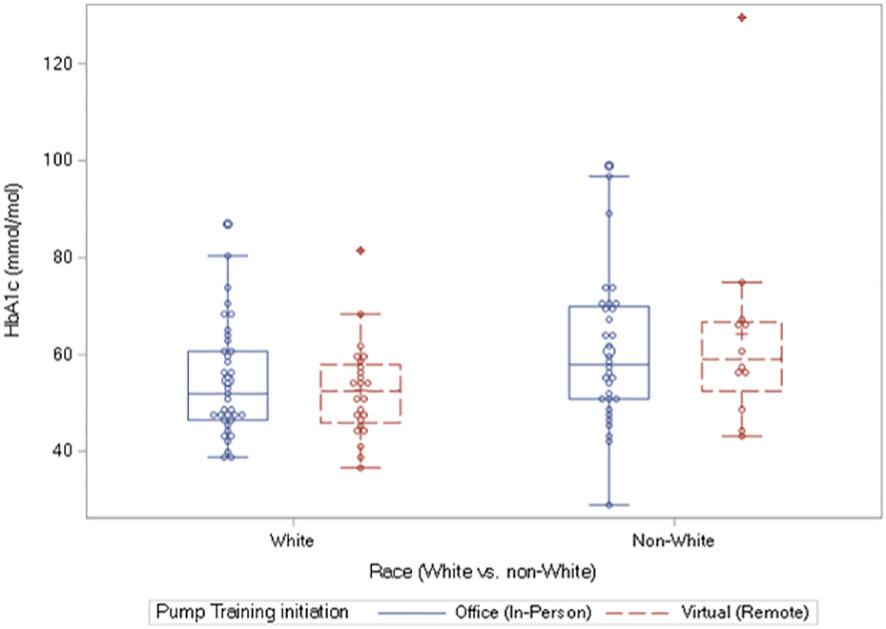
Box plots of A1c values at 6 months showing significantly elevated A1c in the non-White individuals versus White individuals: 57.9 mmol/mol (50.8 – 69.4) versus 51.9 mmol/mol (46.5 -59.6)], p = 0.007. The equivalent results in percent A1c are as follows: 7.45% (6.8-8.5%) versus 6.9% (6.4-7.6%), p = 0.007.

There was no significant association between A1c or TIR with either sex or race. There was equally no relationship between the age at insulin pump initiation and BMI. However, the comparisons of the differences in the markers of glycemic control, A1c and TIR, among the races: White individuals versus non-White individuals, showed a significantly elevated A1c in the non-White individuals at 6 months only: [7.45% (6.8-8.5) versus 6.9% (6.4-7.6)], p = 0.007; [57.9 mmol/mol (50.8 – 69.4) versus 51.9 mmol/mol (46.5 -59.6)], p = 0.007 (**Figure 2**).

### Safety

Over the 6 months of observation, there were no instances of severe hypoglycemia or diabetic ketoacidosis in either group. Specifically, there was no difference in severe hypoglycemia marked by time below range (TBR) of < 54 mg/dL, or severe hyperglycemia marked by time above range (TAR) of >250 mg/L, or glucose variability (GV) between the groups at 0, 3, and 6 months. However, mild hypoglycemia, marked by TBR of >54 to <70 mg/dL was significantly higher at baseline in the virtual group versus the in-office group: 1.02% (1.0 - 2.0), versus 1.0% (0.0 - 1.0), p = 0.004, but was similar between the two groups at 3 and 6 months.

## Discussion

We report that virtual initiation of insulin pumps is safe and effective in children and adolescents with type 1 diabetes. There were no significant differences in A1c, TIR, TAR, GV, and GMI during the period of observation. Furthermore, there were no associations between these parameters with age or sex. However, A1c was significantly elevated at 6 months in the non-White children compared to the White children. Given that TIR and GMI were similar between the racial groups at 6 months, the higher A1c level in non-White children at 6 months could represent the onset of poorer glycemic control or the phenomenon of higher glycation index in US minority population who have a higher mean A1c value of 0.4% for the same mean glucose concentration as their White peers (11).

The subjects who started on insulin pump therapy remotely were all on Omnipod EROS and DASH which do not have an integrated algorithm. Despite this limitation, these subjects performed comparatively well, similar to those who used algorithms, as shown by the lack of significant differences in the parameters of glycemic control in the intergroup analysis. Further intergroup analysis of subjects who used only Omnipod insulin pump in either group showed no significant differences between the two groups. This is consistent with the report that the primary driver of improved glycemia in subjects using diabetes technology is the CGM (9, 12).

This study shows that diabetes technology closed the gap in glycemia between White and non-White children and adolescents in the short term, indicating that access to diabetes technology has the potential to bridge the gap in glycemic outcomes in the long term and reduce the healthcare burden of diabetes management in vulnerable populations. It also showed that both groups were astute in the understanding and use of diabetes technology. There were no significant differences in short-term complications such as hypoglycemia, hyperglycemia, and diabetic ketoacidosis between the White and non-White subjects, suggesting that diabetes technology is well utilized by both groups.

This finding is reassuring and suggests that virtual or remote insulin pump initiation could serve as a tool for optimal diabetes management in future pandemics. These results point to innovative approaches for the adoption and retention of diabetes technology in certain subsets of children with type 1 diabetes where access to diabetes technology is limited. The safety and efficacy data of virtual insulin pump initiation will enable children living in remote parts of the country, where access to pediatric endocrinology clinics is limited, to access diabetes technology to ensure optimal diabetes care. The strong correlation between GMI and A1c suggests a potential role for telemedicine in type 1 diabetes management as insulin adjustments could be conducted using TIR, GMI, and CGM mean glucose information as clinical guides. This approach will improve engagement with medication administration, clinic visits, and continuity of care.

Our study and others show a steep discrepancy in the adoption and use of diabetes technology between the White subjects and non-White populations (13, 14). The reasons for this discrepancy range from unconscious bias from healthcare providers to lack of transportation and means of accessing diabetes technology (15, 16). The proof of safety and efficacy of virtual pump initiation will demystify the process of insulin pump initiation and allow this technology to be made widely available to all sectors of the population.

This study has several limitations. It is a retrospective study, so no causality is inferred in the results. We did not have Tanner stage information to include in our model, however, age correlates closely with Tanner stage. We cannot exclude the possibility of a selection bias in the cohort that favored children from families with higher academic achievement. The proportion of Black children in the study is rather small compared to White children. However, this represents the prevalence rates of type 1 diabetes in different populations, as well as the gap in the adoption and retention of diabetes technology in children with diabetes mellitus, which this work aims to address.

The strengths of this study include a representative sample of virtual and in-person insulin pump initiation which allowed for stratifications; a reasonable follow-up period of six months to establish safety and efficacy; and the use of various glycemic markers such as A1c, GMI, and TIR to demonstrate efficacy.

## Conclusions

We report that virtual insulin pump initiation has a similar safety and efficacy profile as an in-office insulin pump initiation in maintaining optimal glycemia in children and adolescents with type 1 diabetes. This study further demonstrates that diabetes technology closed the gap in glycemia between the White and non-White subjects in the short term; and that both groups were astute in the understanding and use of diabetes technology, suggesting that access to diabetes technology has the potential to close the outcome gap in glycemic management in the long term. The strong correlation between GMI and A1c suggests that GMI, TIR, and other CGM metrics such as CGM mean glucose could serve as veritable tools for telemedicine in pediatric diabetes care. These exciting results provide support for a larger randomized controlled trial to demonstrate the non-inferiority of virtual insulin pump initiation versus in-person initiation. This strategy, which takes the technology to the patient, instead of the patient going to the technology, represents a paradigm shift that could accelerate the adoption and retention of diabetes technology in geographically remote parts of the country, and among the minority populations in the US.

## Data availability statement

The raw data supporting the conclusions of this article will be made available by the authors, without undue reservation.

## Ethics statement

The studies involving humans were approved by Institutional Review Board of Northwell Health. The studies were conducted in accordance with the local legislation and institutional requirements. The ethics committee/institutional review board waived the requirement of written informed consent for participation from the participants or the participants’ legal guardians/next of kin because This was a retrospective study.

## Author contributions

BN: Conceptualization, Data curation, Formal analysis, Investigation, Methodology, Project administration, Resources, Supervision, Validation, Visualization, Writing – original draft, Writing – review & editing. MP: Conceptualization, Data curation, Investigation, Project administration, Resources, Validation, Visualization, Methodology, Writing – review & editing. MNP: Data curation, Formal analysis, Investigation, Methodology, Project administration, Resources, Validation, Visualization, Writing – review & editing. JC: Data curation, Investigation, Methodology, Project administration, Resources, Validation, Visualization, Writing – review & editing. RT: Data curation, Investigation, Methodology, Project administration, Resources, Software, Supervision, Validation, Visualization, Writing – review & editing. RS: Data curation, Formal analysis, Methodology, Resources, Software, Supervision, Validation, Writing – review & editing.
